# Genetic variation and clinical phenotype analysis of hypermethioninemia caused by *MAT1A* gene mutation: Case report

**DOI:** 10.1097/MD.0000000000040957

**Published:** 2024-12-20

**Authors:** Jialin Mu, Yulin Li, Meng Sun, Panpan Li, Jingyun Wang, Hui Zou

**Affiliations:** a Newborn Screening Center, Jinan Maternal and Child Care Hospital, Jinan, P.R.China.

**Keywords:** clinical manifestation, genetic variation, hypermethioninemia, *MAT1A*, newborn screening, treatment

## Abstract

**Rationale:**

The high clinical heterogeneity of hypermethioninemia caused by *MAT1A* gene defects has resulted in a paucity of studies examining the association between clinical phenotypes, biochemical characteristics, and gene mutations in this patient group. Furthermore, the indications for therapeutic interventions in patients remain unclear. The objective of this study is to provide a foundation for clinical diagnosis, genetic counseling, and follow-up management of hypermethioninemia caused by *MAT1A* gene defects.

**Patient concerns:**

A retrospective analysis of children with hypermethioninemia at Jinan Maternal and Child Health Hospital from January 2016 to December 2023 was performed using tandem mass spectrometry (MS/MS). The screened and diagnosed children were tested for gene mutations using second-generation sequencing technology and confirmed using Sanger sequencing.

**Diagnoses:**

Newborn MS/MS screening for diseases demonstrated an elevated methionine level, which was outside the reference range. Upon recalling the newborns, the methionine levels remained elevated, necessitating further refinement of genetic testing. Ultimately, genetic testing confirmed hypermethioninemia, which was attributed to a mutation in the *MAT1A* gene.

**Interventions:**

The intervention for the patients in this study took the following forms: regular follow-up without treatment (n = 3), intake of methionine-free milk powder without any medication (n = 4), intake of methionine-free milk powder with some medication, and eventually liver transplantation (n = 1).

**Outcomes:**

A total of 14 mutation types were detected, including 3 compound heterozygous mutation types (c.926G > T, c.37_38delCT, and c.316G > A) that have not been previously reported. One patient had monoheterozygous mutations, including the novel mutation c.550-1G > A. Eight cases were monitored over time, 7 of which demonstrated typical growth and development. One infant with growth retardation was fed a special formula lacking methionine. The patient underwent liver transplantation. Subsequent follow-up examinations showed methionine and homocysteine levels within normal limits and no further neurological manifestations.

**Lessons:**

Compound heterozygous mutations c.874C > T and c.896G > A may result in higher levels of methionine, affecting the central nervous system. For newborns with initial methionine levels of >500 µmol/L, treatment with a low-Met diet is recommended. Liver transplantation may be beneficial for children with severe hypermethioninemia, particularly in preventing central nervous system damage.

## 1. Introduction

Hypermethioninemia is characterized by elevated blood methionine (MET) levels. It can be caused by primary hypermethioninemia, which is divided into methionine degradation processes, including methionine-adenosyltransferase I and III (MAT I/III), glycine N-methyltransferase, and S-adenosylhomocysteine hydrolase (AHCY) deficiencies. Among these, MAT I/III deficiency is the most common.^[1,2]^ Most patients with hypermethioninemia due to MAT I/III deficiencies are asymptomatic. However, a small number of patients may experience growth retardation, a kale-like odor, or neurological abnormalities.^[3]^ It has been demonstrated that even patients with MAT I/III deficiency who do not exhibit clinical signs early in life may develop neurological symptoms later on.^[4]^ MAT I/III deficiency may be linked to neurological symptoms later in life. Therefore, the long-term prognosis of patients with MAT I/III deficiency remains unclear. Few studies have been conducted on this disease, both domestically and internationally, and even fewer studies have evaluated the correlation between clinical phenotypes, biochemical characteristics, and gene mutations in patients with hypermethioninemia caused by MAT I/III deficiency. This study aimed to summarize the prevalence of hypermethioninemia in Jinan. We translated the clinical data of 10 patients with hypermethioninemia due to *MAT1A* gene mutation from our center, which provides a basis for clinical diagnosis, genetic counseling, and follow-up management.

## 2. Materials and methods

### 2.1. Patients

This was a retrospective analysis of the data of children with hypermethioninemia who attended Jinan Maternal and Child Health Hospital from June 2016 to December 2023 and were diagnosed and confirmed by newborn screening. The clinical data of 10 children with hypermethinemia due to *MAT1A* gene mutations were summarized, including sex, newborn screening and review results, genetic test results, and follow-up data collected on clinical manifestations, physical examination (height and weight), treatment, tandem mass spectrometry (MS/MS) test results, and blood homocysteine results. Eligible patients were those who attended Jinan Maternal and Child Health Hospital from June 2016 to December 2023 and were diagnosed and confirmed by newborn screening. Patients were excluded from the study on the grounds that more than 5% of their individual data were missing. This study was approved by the medical ethics committee of Jinan Maternal and Child Care Hospital (Approval No. 2023-1-061)

### 2.2. Methodology

#### 2.2.1. Mass spectrometry

MS/MS analysis was performed using the NeoBase™ Nonderivatized MSMS Kit (PerkinElmer, Wallac Oy, Turku, Finland). An area of 3.2 mm diameter spot (equal to 3.2 µL of blood) was taken from each DBS into a U-96-well microplate. Then, 100 µL of the extract solution containing internal standards was added to each well. After incubation in a plate shaker for 45 minutes (45°C, 700 rpm), 75 µL supernatant was transferred to a fresh V-96-well microplate. Three levels of internal quality control (blank, low, and high) were used for quality control. MET levels were quantitatively analyzed using MS/MS (Xevo TQD, Applied Biosystems, Waters, Milford). The analysts checked whether the quality control and total ion chromatogram were normal and uploaded them to the Anaconda system to analyze the resulting data.

#### 2.2.2. Plasma total homocysteine

Plasma concentrations were determined via an enzymatic cycling method using an automated automatic biochemical analyzer (Olympus AU5400, Shinjuku, Tokyo, Japan). Total homocysteine levels were considered elevated at concentrations above 15 μmol/L.

#### 2.2.3. Whole-exome sequencing analysis

Blood samples were collected from the children and their parents; 2 mL of peripheral venous blood was collected and stored at 4°C in blood collection tubes containing ethylenediaminetetraacetic acid anticoagulant. Genomic DNA was extracted from the blood samples using the salting-out method. DNA concentration was measured using a Sim-100 Ultra-Micro UV Spectrophotometer, after which the DNA was sequenced after quality control. Sequencing of the children’s DNA samples was performed using a NextSeq 500 High-Output Kit (300 cycles) and a NextSeq 500 sequencer. Sanger sequencing was performed to validate the whole-exome sequencing results of the children and their parents. To determine whether the detected mutation sites were reported mutation sites, we searched the Thousand Genomes Database, National Heart, Lung, and Blood Institute Exome Sequencing Database, and Exome Integration Database. The pathogenicity of the detected mutations was determined according to the standards and guidelines for the interpretation of sequence variants: a joint consensus recommendation developed by the American College of Medical Genetics and Genomics to identify reported mutated loci.

## 3. Results

### 3.1. Prevalence of hypermethioninemia

A total of 478,068 newborns were screened by MS/MS at our center, and 6 children with hypermethioninemia were finally diagnosed, giving a majority of hypermethioninemia of 1:79,678 in Jinan City, Shandong Province.

### 3.2. Biochemical features of MAT I/III deficiency

All 10 children had elevated MET on neonatal MS/MS screening, of which 2 (S6 and S9) were diagnosed at our center, and the remaining 8 cases were from various cities and towns in the province (Table 1). Seven of the children were male, 3 were female, and S2 and S3 were twins. The initial MS/MS screening MET level was 334.40 ± 180.90 µmol/L, and the review MET level was 527.61 ± 320.96 µmol/L. Homocysteine levels were elevated in S1 to S4, S9, and S10, with levels of 35.5 ± 13.0 µmol/L. The MS/MS initial screening MET level was 334.40 ± 180.90 µmol/L, and the MS/MS review MET level was 527.61 ± 320.96 µmol/L. Numbers 5 to 8 had an average homocysteine level (<15 µmol/L).

**Table 1:** Screening and review results of 10 cases with MAT I/III deficiency.

Sample ID	Sex	Screening results	Review results	
		MET (µmol/L)	MET (µmol/L)	HCY (µmol/L)
S1	F	130.00	240.00	23.6
S2*	M	468.99	633.66	22.2
S3*	M	466.13	661.62	19.4
S4	F	526.16	771.05	47.9
S5	M	661.00	346.32	12
S6	F	70.27	193.87	7.4
S7	M	230.69	386.54	10.3
S8	M	200.05	124.30	14.89
S9	M	245.60	677.10	36.6
S10	M	345.10	1241.64	51.6

F = female, HCY = homocysteine, M = male, MAT I/III = methionine-adenosyltransferase I and III, MET = methionine.

* 2 and 3 are twins.

### 3.3. *MAT1A* gene testing and family analysis

**Figure 1.**
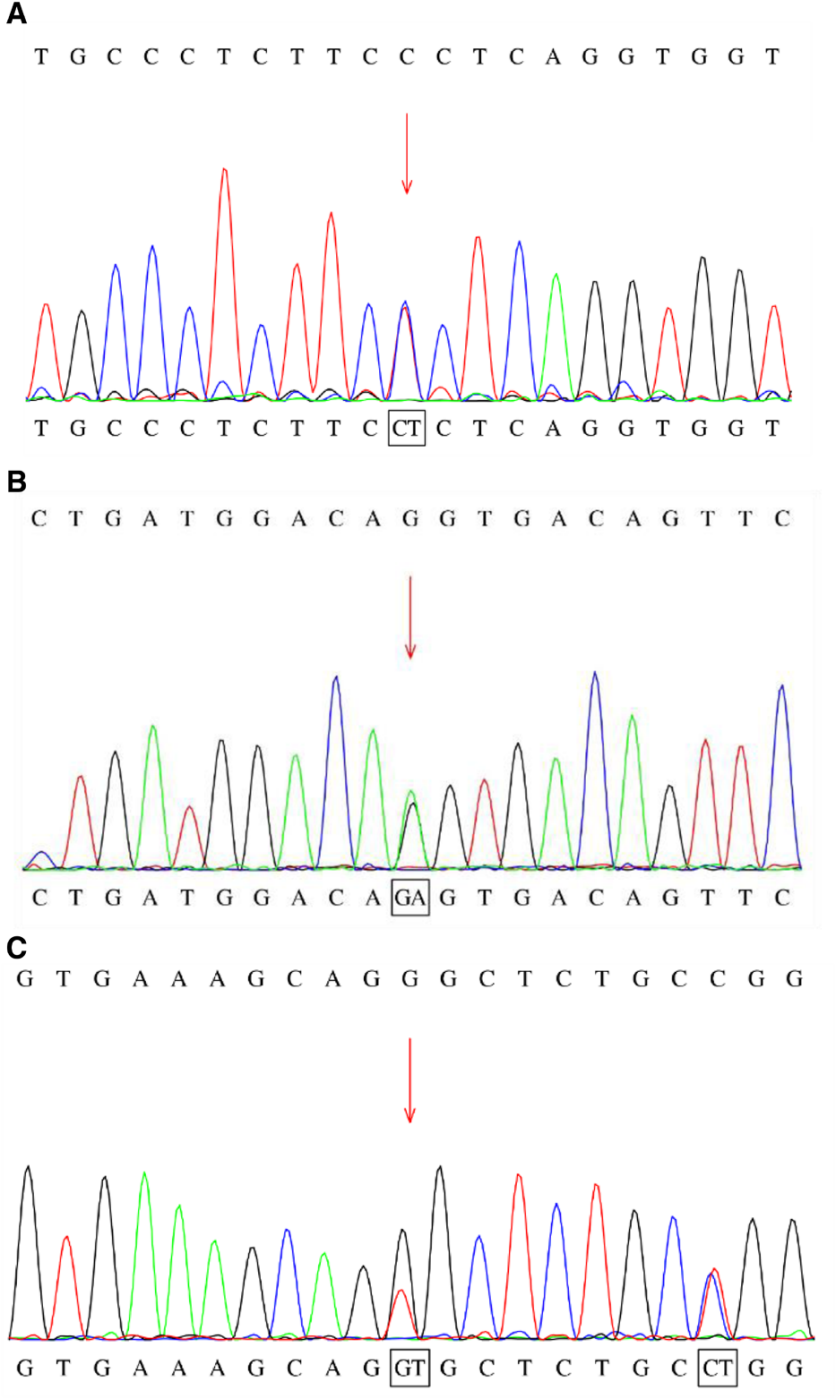
Sequencing map of MAT1A gene mutation sites.

Ten children had mutations in *MAT1A* gene, 6 cases (S5–S10) had compound heterozygous mutations, and 4 cases (S1–S4) had heterozygous mutations (Table 2). Fourteen mutation types were detected: 12 mutations were detected in children with compound heterozygous mutations, 11 types were point mutations, 1 type was a splicing mutation, and 2 types were seen in children with monoheterozygous variants. Of the 12 mutation types associated with compound heterozygosity, 3 have not been reported, namely c.926G > T, c.37_38delCT, and c.316G > A (Fig. 1). Among the cases with monoallelic mutations, 3 (S1–S3) had c.791G > A, a common mutation site, and S4 had c.550-1G > A, a place yet to be reported.

**Table 2:** Results of *MAT1A* gene testing in 10 cases.

Sample ID	Genetics	Exon/intron	cDNA change	Amino acid change	Heterozygosity	Pathogenicity	Inheritance pattern
S1	*MAT1A*	Exon7	c.791G > A	p.R264H	Het	Pathogenic	Maternal
S2*	*MAT1A*	Exon7	c.791G > A	p.R264H	Het	Pathogenic	Paternal
S3*	*MAT1A*	Exon7	c.791G > A	p.R264H	Het	Pathogenic	Paternal
S4	*MAT1A*	-	c.550-1G > A	-	Het	Likely	Paternal
S5	*MAT1A*	Exon3	c.188G > T	p.G63V	Het	Likely	Paternal
	*MAT1A*	Exon7	c.836G > T	p.G279V	Het	VUS	Maternal
S6	*MAT1A*	Exon3	c.242G > A	p.R81Q	Het	VUS	Maternal
	*MAT1A*	Exon4	c.315C > A	p.N105K	Het	VUS	Paternal
S7	*MAT1A*	Exon7	c.934C > T	p.R312W	Het	Likely	Maternal
	*MAT1A*	Exon7	c.926G > T	-	Het	VUS	Paternal
S8	*MAT1A*	Exon6	c.745C > T	p.R249W	Het	Likely	Paternal
	*MAT1A*	Exon1	c.37_38delCT	p.L13Kfs*2	Het	VUS	Maternal
S9	*MAT1A*	Exon7	c.790C > T	p.R264C	Het	Pathogenic	Paternal
	*MAT1A*	Exon4	c.316G > A	p.V106M	Het	VUS	Maternal
S10	*MAT1A*	Exon7	c.896G > A	p.R299H	Het	Pathogenic	Paternal
	*MAT1A*	Exon7	c.874C > T	p.R292C	Het	Likely	Maternal

“-” indicates unspecified.

VUS = variant of uncertain significance.

* Cases 2 and 3 are twins.

### 3.4. Treatment and follow-up

Eight cases (except S2 and S3) were followed up for an extended period, and 5 were fed a low-Met diet (for which the ratio of special nutritional powders to common formula powders was 1:1). The MET and homocysteine levels at follow-up are shown in Table 3. Seven of the 8 children had normal growth and development levels, and 1 child (S10) had a backward story.

**Table 3:** The results of the follow-up data in 8 cases with hypermethioninemia.

Sample ID	Treatment		Relevant indicators		Growth	Intellectual development
	Low-MET diet	Drugs	MET (min~max)	HCY (min~max)		
S1	N	N	94.26–184.67	3.45–10.76	Normal	Normal
S4	Y	N	220.59–393.93	6.30–22.66	Normal	Normal
S5	Y	N	296.99–703.22	4.73–12.56	Normal	Normal
S6	N	N	49.05–149.25	/	Normal	Normal
S7	N	N	38.92–192.51	11.0	Normal	Normal
S8	N	N	47.34–199.84	3.35–12.45	Normal	Normal
S9	Y	N	245.6–423.8	22.1–36.6	Normal	Normal
S10	Y	Y	729.19–1105.17*	41.7*	Normal	Neurological manifestations with developmental lag

“/” represents lack of clinical information.

HCY = homocysteine, MET = methionine, N = none, Y = yes.

* Pretransplant outcome.

S10 was fed a low-Met diet and followed up to 4 months of age. Symptoms such as refusal to eat, drowsiness, and poor mental response are accompanied by involuntary convulsions in the hands and feet. This situation can be alleviated on its own. An EEG test showed abnormalities in the infant’s EEG, with bilateral discharges in the frontal, frontal, anterior temporal, and midline (Fz) regions. At the age of 1.42 years, the baby had received liver transplantation treatment at another hospital. Afterward, he changed to a normal diet, and his MET and homocysteine levels were normal. No further hand and foot convulsions, drowsiness, or adverse psychological reactions were observed.

## 4. Discussion

Hypermethioninemia, a rare amino acid metabolic disease, has a reported prevalence of 1/30,000 to 1/105,000 in foreign countries.^[5,6]^ The prevalence of hypermethioninemia ranges from 1/30,000 to 1/105,000 individuals. In China, the reported majority of hypermethioninemia cases were 1/106,349 in Taiwan Province, 1/116,161 in Zhejiang Province, and 1/116,161 in Shanxi Province.^[7,8]^ The disease was detected in 1/43,279 newborns screened in Shanxi Province, 1/11,469 in Shijiazhuang City, Hebei Province, and 1/27,228 in Henan Province.^[9]^ The prevalence of the disease among newborns screened in the rest of China has not been reported.^[10]^ We summarize the results of newborn screening of 478,068 infants in Jinan City, Shandong Province. Of these, 6 cases were diagnosed with hypermethioninemia, resulting in a prevalence of 1:79,678.

The metabolic pathway of MET in the body involves transsulfuration and transamination. Transsulfuration produces S-adenosylmethionine from MET in the presence of MAT I/III, which is converted to S-adenosylhomocysteine in the presence of glycine N-methyltransferase. Finally, homocysteine was produced in the presence of AHCY.^[11]^ Approximately 50% of patients with simple hypermethioninemia have MAT I/III deficiency caused by *MAT1A* gene mutations. Mutations in *MAT1A* typically exhibit an autosomal recessive inheritance pattern, although some may exhibit autosomal dominant inheritance. Autosomal dominant inheritance is thought to occur because MATI/III is usually present in the tetramer form in the adult liver. Mutated subunits may interfere with related normal subunits, impairing enzyme activity, and resulting in clinical phenotypes in heterozygous carriers. Current evidence indicates that c.791G > A and c.776C > T are the most common heterozygous mutations. These mutations are consistent with an autosomal dominant inheritance pattern, but usually do not adversely affect patients and can be classified as benign variants.^[12]^ Additionally, c.745C > T conforms to an autosomal dominant inheritance pattern and has been shown to affect MAT I/III enzyme activity. However, the enzyme activity was maintained at approximately 80%. It is challenging to identify hotspot mutations in patients with compound and pure heterozygous mutations as they mostly report different mutation sites. Additionally, autosomal recessive patients exhibit greater phenotypic and genetic heterogeneity than do those with autosomal dominant inheritance, making it challenging to predict the relationship between their clinical phenotypes and genotypes. According to previous reports, the c.896G > A and c.874C > T mutations follow an autosomal recessive inheritance pattern. Proteomic studies have confirmed that c.896G > A severely affects the enzyme activity. Children carrying the c.934C > T mutation exhibit severe hypermethioninemia and central nervous system impairment.^[13,14]^ Modifications at c.934C > T significantly affected the enzyme activity. Fourteen mutation types were identified. Two types were detected in children with single heterozygous mutations: c.791G > A and c.550-1G > A variants. None of the 3 cases carrying c.791G > A exhibited any clinical manifestations, which is in agreement with the previous section.^[12]^ S4, which was positive for the c.550-1G > A variant, exhibited hypermethioninemia without any clinical manifestations. This manifestation has not been previously reported, and it is presumed that the c.550-1G > A variant is benign. Six patients with compound heterozygous mutations were examined in this study. S10 carried c.896G > A and c.874C > T compound heterozygous mutations in their parents. He had an initial MET level >1000 µmol/L, which remained poorly controlled even after dietary restriction. At 4 months age of 4 months, the child showed neurological damage. Therefore, it is hypothesized that c.874C > T significantly affects enzyme activity, and that complex heterozygosity with c.896G > A may result in higher MET concentrations. S7 carried compound heterozygous mutations c.934C > T and c.926G > T, of which c.926G > T is an unreported locus. The child had mildly elevated MET levels without abnormalities and had a normal diet, good growth, and development at the later follow-up. The children’s mutation loci were inherited from their parents, consistent with autosomal recessive inheritance. Clinical data suggests that the c.926G > T mutation may be benign. S8 contains compound heterozygous mutations c.745C > T and c.37_38delCT. The c.37_38delCT locus has yet to be reported, and the child inherited the locus variants from their parents. In this case, the child’s MET level was slightly elevated with no apparent clinical manifestations. It is necessary to verify the protein function and inheritance pattern of the c.37_38delCT locus. Here, we report 4 newly identified *MAT1A* mutations that enrich the genetic database. However, more cases are needed to further evaluate the relationship between the genotype and phenotype.

Elevated homocysteine levels may not always be accompanied by simple hypermethioninemia. However, severe MAT I/III defects can result in the persistent elevation of homocysteine levels, which are positively correlated with blood MET levels. This mechanism is due to the reduced blood S-adenosylmethionine levels affecting cystathionine β-synthase activity, which reduces the conversion of homocysteine to cystathionine, leading to elevated homocysteine levels.^[15,16]^ In this study, hyperhomocysteinemia was present in 6 of the 10 children. Of these, 5 had mildly elevated blood homocysteine levels exceeding 50 µmol/L, and S10 had significantly elevated MET levels. Four patients with normal homocysteine levels had mildly to moderately elevated MET concentrations at the initial and repeated examinations, consistent with the previous section. Therefore, when treating patients with elevated blood MET levels associated with elevated homocysteinemia, it is essential to distinguish between simple hypermethioninemia and homocysteinemia. In patients with homocysteinemia and methioninemia, it is essential to differentiate between cystathionine synthase and MAT I/III deficiencies. Measurement of plasma S-adenosylmethionine levels can aid in this differentiation.^[17]^

Patients with mild-to-moderate hypermethioninemia, typically diagnosed through newborn screening, do not exhibit any clinical symptoms and do not require therapeutic intervention.^[18]^ The indications for a low-Met diet treatment remain a matter of debate. The indications for low-Met diet treatment remain a matter of discussion. It is generally accepted that central nervous system damage is more likely when blood MET levels exceed 800 or 900 µmol/L after 5 months of age. In such cases, severe dietary restrictions are recommended. Chien^[19]^ recommended initiating dietary treatment in patients with mean MET levels exceeding 500 to 600 µmol/L. The presumption was that there were no CNS abnormalities. However, case reports have shown no abnormal neurological manifestations with a restricted diet. MET concentrations increased rapidly after the mice were fed a normal diet, and abnormal magnetic resonance imaging findings were observed. Hirabayashi et al^[20]^ reported a case in which a patient with MAT I/III deficiency and neurological involvement improved after low-Met diet treatment. These findings suggest that restricting MET intake has protective and therapeutic effects on the central nervous system. This study included 8 children who were followed up for an extended period. Seven patients did not exhibit any clinical manifestation. Four patients with MET levels >500 µmol/L were given a low-Met diet treatment (for which the ratio of special nutritional powders to common formula powders was 1:1), and their MET levels decreased significantly during follow-up compared to the screening and review results. The other 2 children had a screening or review MET level <500 µmol/L, and their MET levels also significantly decreased during follow-up after being fed a normal diet. None of the patients showed clinical symptoms, and their growth and development were average in the later stages. A low-Met diet can help control MET levels, prevent damage to the central nervous system, and alleviate family anxiety. MET concentrations above 500 to 600 µmol/L can be treated with dietary regimens, as appropriate.

Pediatric liver transplantation is indicated for metabolic diseases, as the liver plays a central role in the body’s transsulfuration-methyl metabolic pathway. The primary benefit of liver transplantation in children with metabolic diseases is to correct metabolic disturbances to prevent neurological damage and ensure long-term survival, unlike in children undergoing liver transplantation for end-stage liver disease.^[21]^ Liver transplantation is a rare treatment option for children with hypermethioninemia; however, the indications for liver transplantation in this population are unclear. This case report describes a child with AHCY deficiency and hypermethioninemia, who presented with generalized seizures at 8 months of age. The child’s MET level was 614 µmol/L, and magnetic resonance imaging revealed demyelination in the brain. Despite the dietary treatment, the child’s condition remained poorly controlled. After liver transplantation, MET levels were normalized. Magnetic resonance results indicated a reduction in the degree of demyelination. Neurological symptoms were observed.^[22]^ In this study, a child with a blood MET level >1000 µmol/L was given special milk powder to remove the MET. The milk powder was mixed proportionally, and the child was followed up until the age of 4 months when neurological damage occurred. The MET level remained elevated and a complete electroencephalogram indicated abnormal brain activity in the infant. Special milk powder was administered to increase the dosage, but hand and foot convulsions persisted. The patient underwent liver transplantation at an external hospital, after which there were no further neurological symptoms. On follow-up, the patient’s methionine and homocysteine levels were normal, and their growth was average with a slight developmental delay. These findings confirm that liver transplantation can be beneficial in children with neurological damage and hypermethioninemia. Unfortunately, multiple refinements of cranial magnetic resonance examinations in this case were unsuccessful and cranial involvement could not be clarified. Liver transplantation may be beneficial for children with hypermethioninemia levels exceeding 1000 µmol/L at an early age. However, further research is needed to determine the long-term complications and survival rates of these patients.

The limitations of this study include the fact that all of the children with hypermethioninemia diagnosed by neonatal screening at our center were below the age of adolescence. Furthermore, the long-term prognosis of patients with simple hypermethioninemia remains unclear. The *MAT1A* gene mutations result in multisystemic damage, particularly to the central nervous system and other systems. Further investigation is required to elucidate this phenomenon.

## 5. Conclusion

Newborn screening programs can effectively detect hypermethioninemia. Hyperhomocysteinemia caused by mutations in the *MAT1A* gene can be diagnosed by combining relevant biochemical indices with the results of genetic testing. The clinical presentation and biochemical indices of this condition show some degree of heterogeneity, which may be related to the degree of expressed enzyme activity. For newborns with initial MET levels of >500 µmol/L, treatment with a low-Met diet is recommended. Liver transplantation is not the standard treatment for patients with hypermethioninemia. However, early liver transplantation can provide significant benefits in cases of severe hypermethioninemia. It is essential to understand the indications for transplantation and ensure an excellent long-term follow-up. The long-term prognosis of patients with simple hypermethioninemia due to *MAT1A* gene mutations, particularly damage to the central nervous system, remains challenging. Further research is required to thoroughly understand this issue.

## Author contributions

**Conceptualization:** Jialin Mu, Hui Zou.

**Data curation:** Jialin Mu, Yulin Li, Panpan Li, Jingyun Wang.

**Formal analysis:** Jialin Mu, Yulin Li, Hui Zou.

**Investigation:** Jialin Mu, Yulin Li.

**Methodology:** Jialin Mu, Yulin Li.

**Writing—original draft:** Jialin Mu.

**Project administration:** Meng Sun.

**Resources:** Meng Sun.

**Software:** Meng Sun.

**Funding acquisition:** Hui Zou.

**Writing—review & editing:** Hui Zou.
